# Linc00483 as ceRNA regulates proliferation and apoptosis through activating MAPKs in gastric cancer

**DOI:** 10.1111/jcmm.13661

**Published:** 2018-05-15

**Authors:** Defeng Li, Meifeng Yang, Aijun Liao, Bing Zeng, Diqun Liu, Yuhong Yao, Guangsheng Hu, Xuanmin Chen, Zhiqiang Feng, Yanlei Du, Youlian Zhou, Jie He, Yuqiang Nie

**Affiliations:** ^1^ Department of Gastroenterology and Hepatology Guangzhou Digestive Disease Center Guangzhou First People's Hospital Guangzhou Medical University Guangzhou China; ^2^ Guangzhou Key Laboratory of Digestive Disease Guangzhou First People's Hospital Guangzhou China; ^3^ Department of Gastroenterology The Second Affiliated Hospital Medical School South China University of Technology Guangzhou China; ^4^ Department of Gastroenterology The First Affiliated Hospital of South China of University South China of University Hengyang China; ^5^ Department of Hematology The First Affiliated Hospital of South China of University South China of University Hengyang China

**Keywords:** ceRNA, gastric cancer, linc00483, MAPKs, miR‐30a‐3p, SPAG9

## Abstract

Long non‐coding RNAs (lncRNAs) are important regulators of many cellular processes, and their aberrant expression and/or function is associated with many different diseases, including cancer. However, the identification of functional lncRNAs in gastric cancer is still a challenge. In this study, we describe a novel functional lncRNA, linc00483, that is upregulated and associated with tumorigenesis, tumour size, metastasis and poor prognosis in gastric cancer. In our study, linc00483 promoted gastric cancer cell proliferation, invasiveness and metastasis in vitro and in vivo. Mechanistically, upregulated expression of linc00483 in gastric cancer acts as a sponge to absorb endogenous tumour suppressor miR‐30a‐3p. Furthermore, it restores SPAG9 expression, which is negatively regulated by miR‐30a‐3p, and actives MAPK signaling pathway in gastric cancer cells. Thus, linc00483 is an oncogenic lncRNA in gastric cancer and targeting linc00483 or its pathway can potentially be useful in development of targeted therapies for patients with gastric cancer. Our results show that linc00483 is an important regulator in carcinogenesis and may be a useful biomarker to predict prognosis of gastric cancer patients. We believe our findings are novel and will be of interest to scientists working in many areas related to biomarkers in cancer.

## 1 INTRODUCTION

Gastric cancer is a serious health and economic problem worldwide, and according to the GLOBOCAN 2015 reports, gastric cancer is the third leading cause of cancer‐related deaths globally, with the majority of patients reported in developing countries.^1^ Most gastric cancer patients are diagnosed at an advanced stage, and thus, the 5‐year survival rate is low.^2^ Improved and early diagnosis as well as better targeted therapies are required. In line with this is the importance of identifying genes that contribute to gastric cancer development and have clinical predictive value for patients’ diagnosis or prognosis. Gastric cancer, like most malignant tumours, exhibits common characteristic hallmarks of cancer including increased proliferation and inhibition of apoptosis. Currently, many potential biomarkers have been reported for gastric cancer, such as CEACAM6, HER2, MFSD4 and MET,^3^, ^4^, ^5^, ^6^ but only modest successes have been obtained in their translation to the clinical setting.

The human genome encodes more than 10 000‐long non‐coding RNAs (lncRNAs) that have no protein‐coding capacity.^7^ LncRNAs are a class of transcripts exceeding 200 nucleotides, and research has shown frequent deregulation of lncRNAs in various diseases and multiple functions of lncRNAs in a wide range of biological processes such as cell proliferation, apoptosis and migration.^8^, ^9^, ^10^, ^11^ For example, lncRNA PVT1 expression was reported to be significantly upregulated in non‐small cell lung cancer and was significantly correlated with histological grade and lymph node metastasis. In addition, knockdown of PVT1 reportedly inhibits non‐small cell lung cancer cell proliferation, migration and invasion.^12^ Furthermore, lncRNA BC200 expression was found to be reduced in ovarian cancer and its inhibition was shown to promote the proliferation of SKOV3 and A2780 ovarian cancer cells.^13^ Another study reported that the lncRNA PVT1 level in gastric cancer is significantly higher than levels in adjacent normal tissues and concluded that PVT1 may be a promising biomarker for early detection and prognosis in this type of cancer.^14^ Although a small number of lncRNAs have been functionally characterized, most still remain functionally undefined.^15^, ^16^, ^17^, ^18^, ^19^, ^20^

LncRNAs can also act as decoys for microRNAs to regulate the expression of coding genes by competing endogenous RNAs (ceRNAs).^21^ For instance, in hepatocellular carcinoma, linc00974 was shown to enhance the expression of miR‐642 target gene KRT19 by competitively sequestering miR‐642 and to promote hepatocellular carcinoma cell proliferation.^22^ Furthermore, lncRNA‐ATB can upregulate the expression of ZEB1 and ZEB2 by competitively acting as a decoy for the miR‐200 family and in this way contribute to epithelial‐mesenchymal transition in hepatocellular cancer.^23^ Despite these findings, our knowledge on lncRNA expression and function in tumorigenesis is still limited. More than 65% of lncRNAs are located less than 10  kb from established regulating protein‐coding genes either in a *cis*‐ *or trans*‐regulatory manner.^9^ Linc00974 has been confirmed to be a *cis*‐regulator of the KRT19 gene, promoting proliferation and metastasis beyond the Flank 10 kb class in hepatocellular carcinoma.^22^ In this study, we discovered a novel lncRNA, linc00483, via Affymetrix microarrays.^24^

According to our findings, linc00483 displays considerable predictive value for the diagnosis and prognosis of gastric cancer. Furthermore, linc00483 expression strongly correlated with SPAG9 expression, which is a scaffold protein in the MAPK pathway.^25^ Bioinformatic analyses revealed that linc00483 is located at a locus 20 kb upstream of the SPAG9 gene. Here, we report that linc00483 promotes proliferation and inhibits apoptosis through upregulation of SPAG9 and activation of MAPKs in both an in vitro and in vivo gastric cancer model. We further demonstrate that linc00483 acts as a trap for miR‐30a‐3p, which targets SPAG9 mRNA for degradation. Based on these findings, we propose a model for linc00483‐mediated cell proliferation and apoptosis in gastric cancer.

## 2 MATERIALS AND METHODS

### 2.1 Patient samples

The study included 48 gastric cancer patients recruited between August 2012 and September 2014 in Guangzhou First People's Hospital (Guangzhou, China). A written informed consent was obtained from all patients prior to surgery, and the study was approved by our Institutional Ethics Committee. From each patient, samples of gastric tumour and corresponding normal mucous tissues were taken, and samples were examined by a trained pathologist for confirmation of diagnosis. All research was performed in compliance with government policies and the Declaration of Helsinki.

### 2.2 Cell culture

The human gastric cancer cell lines SGC7901, BGC823, MGC803 and MNK28, and the normal gastric epithelial cell line GES‐1 were obtained from Guangzhou Medical University (Guangzhou, China). Cells were cultured in RPMI1640 Gibco, USA) supplemented with 10% foetal bovine serum (FBS, Gibco, USA) and 1% penicillin/streptomycin in a humidified incubator at 37°C in an atmosphere of 5% CO_2_.

### 2.3 Cell transfection

For the cell transfection experiments, siRNA and miR‐30a‐3p mimics were designed and synthesized by Sangon (Shanghai, China). DharmaFECT^™^ Duo Transfection Reagent was purchased from Dharmacon (NY, USA). Cells were seeded in 96‐well plates at a concentration of 1 × 10^6^ cells per well and transfected with siRNA and miR‐30a‐3p mimics in RPMI‐1640 (Gibco, USA) according to the manufacturer's protocol. After 24 hours, transfected cells were collected and used in further experiments.

### 2.4 Cell proliferation assay

Cell proliferation was assayed using the CCK8 test (Roche, Switzerland) according to the manufacturer's protocol.

### 2.5 Cell invasion assay

For the invasion assay, Transwell plates with inserts were used. The inserts were coated with Matrigel (Shanghai, China) and placed in an incubator to solidify. Next, cells (10^6^ cells per well) were seeded in serum‐free medium in the upper compartment, and culture medium with 10% FBS was added to the lower compartment. After 24 hours, cells from the upper surface of the insert were removed with a cotton swab, and cells on the lower surface of the insert were fixed with 95% alcohol, stained with 0.1% crystal violet and counted under an inverted microscope. Each experiment was performed in triplicate.

### 2.6 Wound healing assay

For the wound healing assay, cells (1 × 10^6^) were seeded in a monolayer on 96‐well plates, and a scratch was made with a new 1‐mL pipette tip across the centre of each well. After 0, 12 and 24 hours, wounds were photographed under an inverted microscope (Thermo, USA) at a magnification of 200×, and measurements were taken.

### 2.7 Luciferase reporter gene assay

For the luciferase reporter gene assay, cells were plated in 24‐well plates. Previous analysis predicted that miR‐30a‐3p interacts with the 3′ UTR sequence of SPAG9. The complete length of linc00483 or its mutated variant with predicted target sites was inserted into the pGL3 promoter vector, and Renilla luciferase vector pRL‐SV40 (5 ng) was cotransfected to normalize the differences in transfection efficiency.

### 2.8 Subcutaneous xenotransplantation model

For the in vivo study, BGC823 cells (1 × 10^7^) were subcutaneously implanted into the bilateral axilla of 18 BALB/C nude mice. When xenotransplantation was established, mice were randomly divided into 3 groups and injected with either linc00483‐siRNA, linc00483‐NC, or NS with Jet‐PET every 2 days. After 2 weeks, tumours were measured every 7 days, and the corresponding volumes were calculated. Mice were sacrificed after 4 weeks, and tumour tissues were used to establish implant models as described previously.

### 2.9 Quantitative real‐time PCR

Quantitative real‐time PCR (qPCR) was performed to determine the expression levels of linc00483, miR‐30a‐3p and SPAG9 mRNAs in gastric tumours and corresponding normal mucous tissues. Total RNA was obtained from tissues or cultured cells using TRizol reagent (Takara, Japan). For extraction of RNA from the cytoplasm and nucleus, the SurePrep Nuclear or Cytoplasmic RNA Purification Kit from Thermo Fisher Scientific (Rochester, NY, USA) was used according to the manufacturer's protocols. For mRNA detection, total RNA was reverse transcribed using the reverse transcription kit (Takara, Japan). qPCR was performed with a Light Cycle 480II (Roche, Switzerland). The primer sequences used in qPCR are listed in Table S3. The miR‐30a‐3p primer was provided by Qiagen (Qiagen, Germany). GAPDH and U6 were used as internal controls.

### 2.10 Immunohistochemistry

For immunohistochemistry, tissue sections (5 μm) were deparaffinized in xylene and rehydrated with a graded ethanol series. Next, slides were treated with 3% H_2_O_2_ in methanol for 30 minutes to block endogenous peroxidase activity. Slides were rinsed in PBS and incubated with 10% normal goat serum for 30 minutes. After washing in phosphate‐buffered saline (PBS), slides were incubated with primary anti‐rabbit antibodies to SPAG9, c‐Jun, p‐Jnk, p53 and p‐p38 (Abcam, UK) at 4°C overnight. The next day slides were washed with PBS and incubated with secondary antibody for 2 hours at room temperature. Finally, slides were stained with DAB, counterstained with haematoxylin, mounted and photographed using a digitalized microscope camera (Nikon, Japan).

### 2.11 Western blot analysis

For western blot analysis, proteins were extracted from tissues or cultured cells using radioimmunoprecipitation assay (RIPA) buffer (Beyotime, China). Total protein extract (40 μg/μL) was loaded onto 12% polyacrylamide gels and after electrophoretic separation transferred to a polyvinylidene difluoride membrane (BiYunTian, China). Next, membranes were blocked with 1 mL TIP and incubated overnight at 4°C with primary polyclonal antibodies against SPAG9, c‐Jun, p‐Jnk, p53 and p‐p38 (Abcam) at 1:500 dilution. Next day, the membranes were washed and incubated with secondary antibody (Abcam) for 1 hour at room temperature. Finally, membranes were visualized using UVP (BioRad, Germany). Equal protein loading in each lane was confirmed using the β‐actin antibody. The integrated density of each band was quantified using Proteinsimple Western (Protein Simple, USA).

### 2.12 Statistical analysis

Statistical analysis was performed with SPSS 18.0 (SPSS, Inc., Chicago, IL, USA). The results of qPCR analysis are presented as mean ± standard deviation (SD). All data were analysed using the Student's *t* test and analysis of variance as appropriate. Pearson correlation analysis was used to analyse the relationship between linc00483, SPAG9 and miR‐30a‐3p expression levels in gastric cancer samples. Receiver operating characteristic (ROC) curve analysis was used to evaluate the diagnostic potential of linc00483 and to find the appropriate cut‐off point. In all cases, *P* < .05 was considered significant.

## 3 RESULTS

### 3.1 Deregulation of lncRNAs in gastric cancer

Six gastric cancer tissues and paired normal tissues were analysed in our previous study to obtain the lncRNAs profiles using the ArrayStar lncRNA microarray in gastric cancer.^24^ According to the results, there were 2541 upregulated and 1281 downregulated lncRNAs according to criteria of *P* < .05 and fold change (FC) > 2.0.^24^ For this study, we selected 5 upregulated and 5 downregulated lncRNAs (*P* < .01, FC > 45) for further analysis of their expression by RT‐qPCR in a larger cohort of gastric cancer patients (n = 48; Table S1). The results of this analysis identified linc00483 upregulation in both gastric cancer and gastric cancer cell lines (Figures S1 and S2). Notably, linc00483 was annotated as a long intergenic non‐protein coding RNA in the UCSC genome database, but its function in gastric cancer remained unknown (Figure S3).

### 3.2 Linc00483 upregulation is associated with shorter survival in gastric cancer patients

In our study, linc00483 expression was higher in gastric cancer tissues than in paired normal gastric tissues by RT‐qPCR (n = 48; Figure S4A). In addition, linc00483 expression was further analysed according to patients’ different clinicopathological features (ie, age, sex, tumour size, differentiation, invasion depth, TNM stages, lymphatic metastasis and distant metastasis). Higher expression of linc00483 correlated with larger tumour size, tumour cell differentiation, greater invasion depth, more advanced TNM stage, lymphatic metastasis and distant metastasis, but was not significantly associated with gender or age (Table S2). According to the mean ratio of relative expression (C/N) and median count, the 48 gastric cancer cases were assigned to either high‐expression (n = 31, >18.22) and low‐expression (n = 17, <18.22) groups. Kaplan‐Meier survival analysis and log‐rank tests showed that higher linc00483 expression was associated with shorter survival time (*P* = .0088; Figure S4B). ROC curves were constructed to evaluate the sensitivity and specificity of linc00483 for prediction of gastric cancer diagnosis. The area under the ROC curve for linc00483 was 0.959 (95% confidence interval = 0.937‐0.995, *P* < .01; Figure S4C). Collectively, these findings indicate that linc00483 is a potential biomarker for diagnosis and prognosis in gastric cancer.

### 3.3 Linc00483 promotes cell proliferation and inhibits apoptosis in vitro

**Figure 1:**
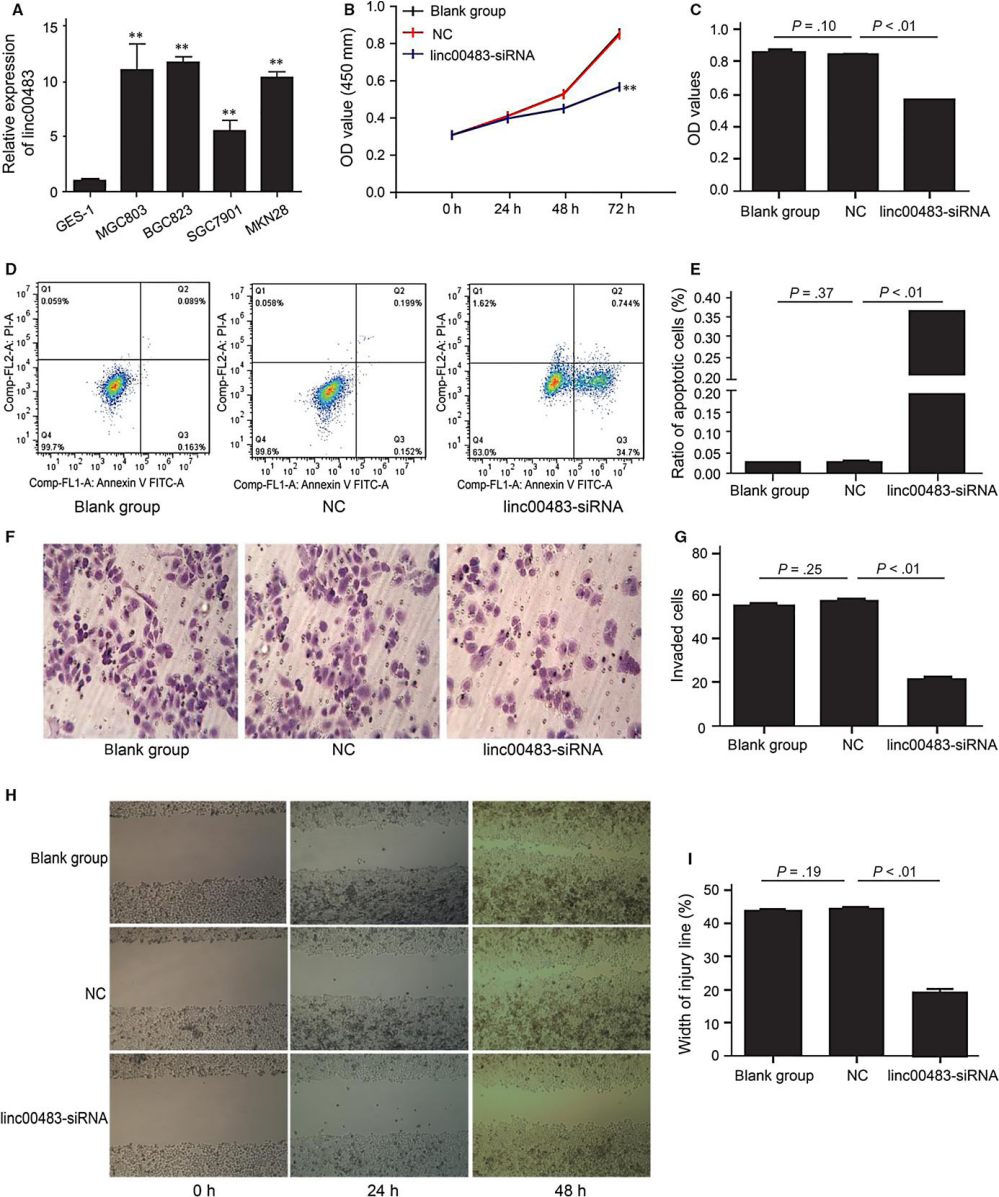
Linc00483 can promote proliferation and inhibit apoptosis of BGC823 cells in vitro. A, Relative expression of linc00483 in human gastric cancer cells and the human immortalized gastric epithelial cell line GES‐1 (*P* < .01). B, C, CCK8 test showed that knockdown of linc00483 results in decreased proliferation of BGC823 cells (*P* < .01). D, E, Flow cytometric analysis showed that linc00483 knockdown increases apoptosis among BGC823 cells (*P* < .01). F, G, Transwell assay indicated that linc00483 silencing can inhibit invasion ability of BGC823 cells (*P* < .01). **P < .01

To examine the biological functions of linc00483, we first detected its expression in human gastric cancer cell lines (SGC7901, MGC803, BGC823 and MKN28) and the human immortalized gastric epithelial cell line GES‐1. Linc00483 expression was upregulated in all gastric cancer cell lines (*P* < .01; Figure 1A), and we selected BGC823 for further study. According to the interference efficiency of candidate shRNAs for linc00483, linc00483‐HOMO‐313 was used for further linc00483 knockdown experiments (Figure S5). The CCK8 assay was used to determine the role of linc00483 in cell growth. In BGC832 cells, linc00483 knockdown led to reduced cell proliferation compared with that observed in the non‐transfected control (NC) cells and cells transfected with blank controls (*P* < .01; Figure 1B,C). Furthermore, annexin V‐FITC/PI was applied to detect apoptosis. Upon linc00483 silencing, the percentage of apoptotic cells was greater than that among NC cells (Figure 1D,E). Furthermore, a Transwell assay revealed that the invasion ability of cells in which linc00483 was silenced was suppressed compared with that of NC and blank control cells (Figure 1F,G). In the wound healing assay, knockdown of linc00483 contributed to slower scratch healing (Figure 1H,I).

### 3.4 Linc00483 expression correlates with SPAG9 mRNA expression

As lncRNAs regulate neighbouring protein‐coding genes in a *cis*‐ or *trans*‐regulatory manner, we examined mRNAs for which expression correlates with linc00483 expression. For this purpose, BGC823 cells with linc00483 knock down, NC and blank control cells were analysed to obtain mRNA profiles using microarray (Figure S6). The results and bioinformatic analyses showed that linc00483 expression correlated with SPAG9 mRNA expression and the linc00483 locus was located 20 kb upstream of SPAG9 (Figure S7A). Next, we examined SPAG9 expression in gastric cancer tissues and paired normal gastric tissues by RT‐qPCR (n = 48), and SPAG9 mRNA expression was higher in gastric cancer tissues than in the corresponding normal gastric tissues (*P* < .01; Figure S7B). In addition, Kaplan‐Meier survival analysis and log‐rank tests showed that patients with higher SPAG9 expression had a poorer prognosis than those with low SPAG9 expression (*P* = .0027; Figure S7C). The Moreso, Pearson correlation assay showed that linc00483 and SPAG9 expression were strongly positively correlated in gastric cancer tissues (*R* = .6170, *P* < .01; Figure S7D). Therefore, we suggested that linc00483 may regulate SPAG9 mRNA expression.

### 3.5 MAPK signalling activation in gastric cancer

**Figure 2:**
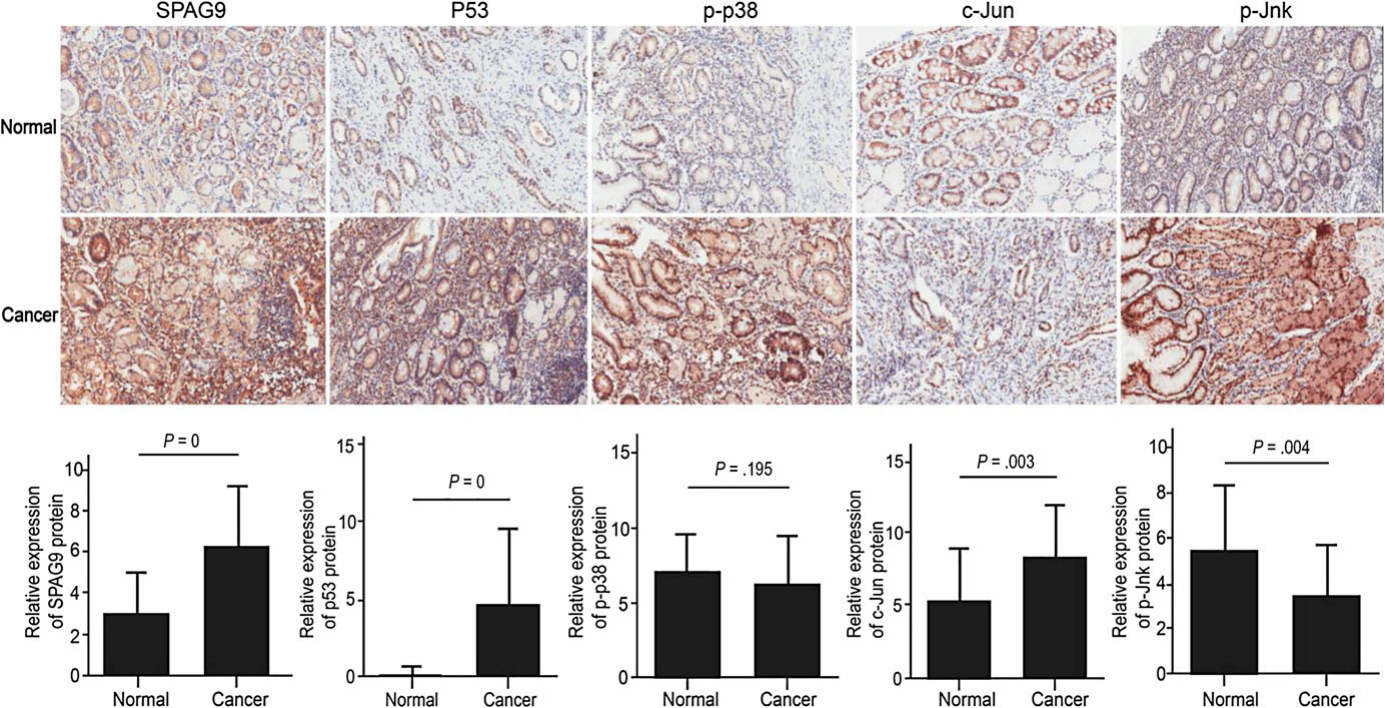
MAPK expression in gastric cancer and normal gastric tissues. The protein expression of SPAG9, p53, p‐p38, p‐Jnk and c‐Jun was examined by immunohistochemistry in 48 paired gastric cancer tissues and normal gastric tissues. SPAG9, p53, c‐Jun and p‐p38 protein expression was significantly higher in gastric cancer tissues than in normal gastric tissues (*P* = 0, *P* = .003 and *P* = 0, respectively), whereas p‐Jnk protein expression was significantly lower in gastric cancer tissues than in normal gastric tissues (*P* = .004). No difference was observed for p‐p38 protein expression (*P* = .195)

To investigate whether MAPK signalling is deregulated in gastric cancer, we examined the expression of SPAG9 and downstream effectors of MAPKs, such as c‐Jun, phosphorylated (p)‐Jnk, p‐p38 and p53, by immunohistochemistry in gastric cancer tissues and paired normal gastric tissues (n = 48). SPAG9, c‐Jun and p53 protein expression was higher in gastric cancer tissues than in paired normal gastric tissues (*P* < .001, *P* = .003 and *P* = .000 respectively). p‐Jnk protein expression was lower in gastric cancer tissues than in paired normal gastric tissues (*P* = .004). p‐p38 expression did not differ between gastric cancer tissues and paired normal gastric tissues (*P* = .195; Figure 2). Thus, based on these results it can be concluded that MAPK signalling is activated in gastric cancer.

### 3.6 Linc00483 regulates SPAG9 and MAPK expression in vitro

**Figure 3:**
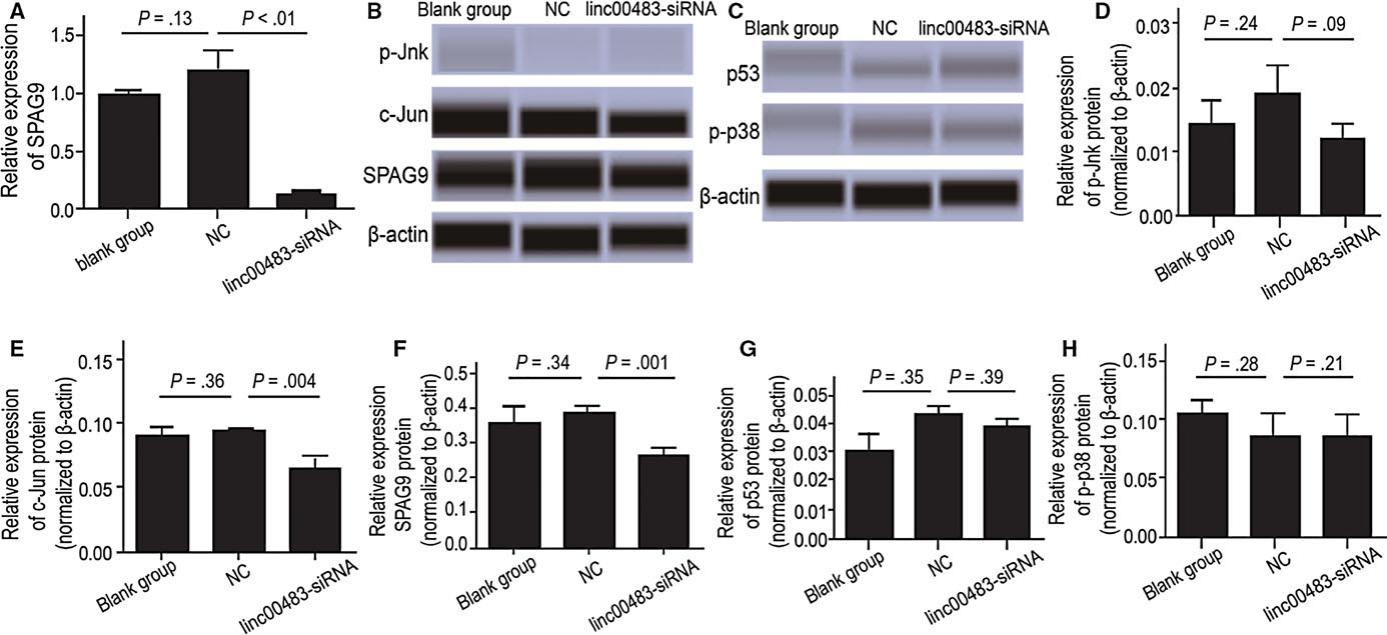
Linc00483 regulates activation of MAPKs in vitro. A, Knockdown of linc00483 can decrease SPAG9 mRNA expression in BGC823 cells (*P* < .05). B, E, F, Suppression of linc00483 can decrease protein expression of SPAG9 and c‐Jun in BGC823 cells (*P* = .004 and *P* = .001, respectively). C, D, H, Suppression of linc00483 does not influence the expression the proteins p53, p‐Jnk and p‐p38 (*P* = .39, *P* = .09 and *P* = .21, respectively)

To explore whether linc00483 regulates SPAG9 and MAPK expression, we silenced linc00483 in BGC823 cells and monitored the effects on SPAG9, c‐Jun, p‐Jnk, p53 and p‐p38 mRNA expression. Linc00483 knockdown substantially decreased SPAG9 mRNA expression as determined by RT‐qPCR (Figure 3A). Consistently, knockdown of linc00483 also decreased SPAG9 and c‐Jun protein expression; however, it did not impair p‐Jnk, p53 and p‐p38 protein levels as determined by western blotting (Figure 3B‐H; *P* = .001, *P* = .004, *P* = .09, *P* = .39 and *P* = .21 respectively).

### 3.7 Linc00483 regulates SPAG9 by acting as a ceRNA and interacting with miR‐30a‐3p

**Figure 4:**
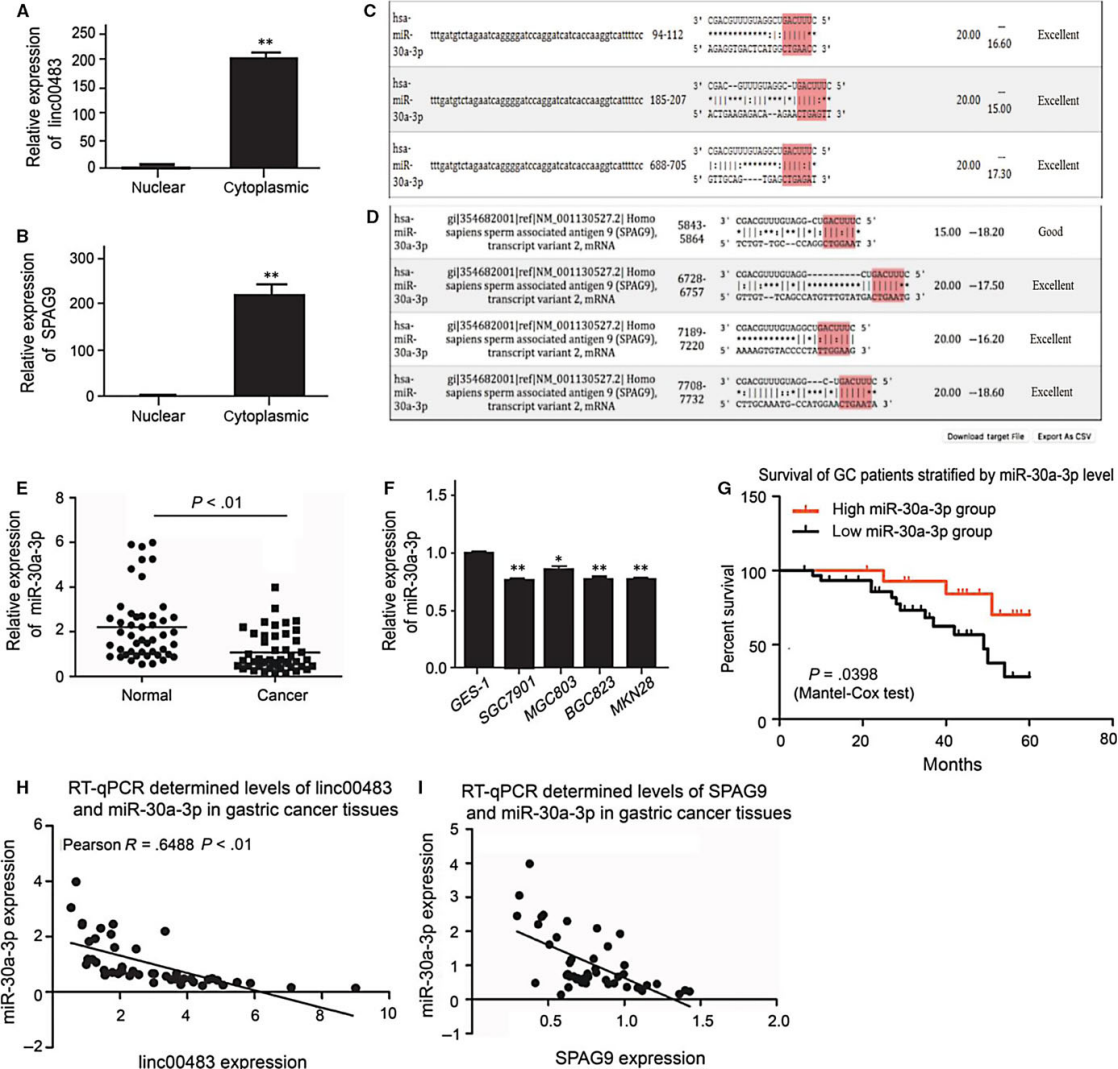
miR‐30a‐3p expression is downregulated and correlates negatively with linc00483 and SPAG9 mRNA expression. A, Linc00483 was detected in cytoplasm by RT‐qPCR after separation of cytoplasmic and nuclear RNA fractions (**P < .01). B, SPAG9 mRNA was detected in the cytoplasm by RT‐qPCR after separation of cytoplasmic and nuclear RNA fractions (**P < .01). C, Bioinformatic analysis showed that miR‐30a‐3p can bind with linc00483. D, Bioinformatic analysis showed that miR‐30a‐3p can bind with SPAG9 3′ UTR. E, Relative expression of miR‐30a‐3p in 48 paired gastric cancer tissues and normal gastric tissues, showing significantly lower miR‐30a‐3p expression in gastric cancer compared to normal gastric tissues (*P* < .01). F, Relative expression of miR‐30a‐3p in human gastric cancer cells and a human immortalized gastric epithelial cell line (GES‐1), showing that miR‐30a‐3p levels were significantly lower in gastric cancer cells (*P < .05, **P < .01). G, Survival time after surgery was compared between patients with high and low miR‐30a‐3p expression by Mantel‐Cox test, and the survival time of patients with high miR‐30a‐3p expression was significantly longer (*P* = .0398). H, Pearson correlation analysis of miR‐30a‐3p and linc00483 expression showed that miR‐30a‐3p expression was negatively correlated with linc00483 in gastric cancer tissue (*R* = −.6488, *P* < .01). I, Pearson correlation analysis of miR‐30a‐3p and SPAG9 mRNA expression showed that miR‐30a‐3p expression negatively correlated with SPAG9 mRNA expression in gastric cancer tissue (*R* = −.6133, *P* < .01)

ceRNAs have microRNA response elements (MREs) that can bind miRNAs, leading to their impairment. ceRNAs are located in the cytoplasm along with the mature microRNAs and RNA‐induced silencing complex.^21^ Therefore, we examined linc00483 and SPAG9 mRNA subcellular localization. First, we separated cytoplasmic RNA from nuclear RNA and then examined their expression in both fractions. The results showed that both linc00483 and SPAG9 are present in the cytoplasm (Figure 4A,B), confirming that linc00483 may regulate SPAG9 mRNA activity as a ceRNA.

After detailed examination of the lncRNA regulatory mechanisms, we conducted a bioinformatic analysis to explore the possible co‐regulation of linc00483 and SPAG9 mRNA. MiR‐30a‐3p was selected as a candidate, as it can bind to both linc00483 and the SPAG9 3′ untranslated region (UTR) (Figure 4C,D). RT‐qPCR analysis showed that miR‐30a‐3p levels were lower in gastric cancer tissues than in paired normal gastric tissues (n = 48) and also in gastric cancer cell lines compared with the GES‐1 cell line (Figure 4E,F). Furthermore, Kaplan‐Meier survival analysis and log‐rank tests showed that patients with higher miR‐30a‐3p expression had longer survival times than those with lower miR‐30‐3p expression (*P* = .0398; Figure 4G). Pearson correlation assay confirmed that miR‐30a‐3p, linc00483 and SPAG9 mRNA were strongly negatively correlated in gastric cancer tissues (*R* = −.6488, *P* < .0001, *R* = −.6133, *P* < .001; Figure 4H,I). Collectively, these results demonstrated that miR‐30a‐3p acts as an anti‐oncogene and plays an important biological function that may correlate with linc00483 and SPAG9 in gastric cancer.

**Figure 5:**
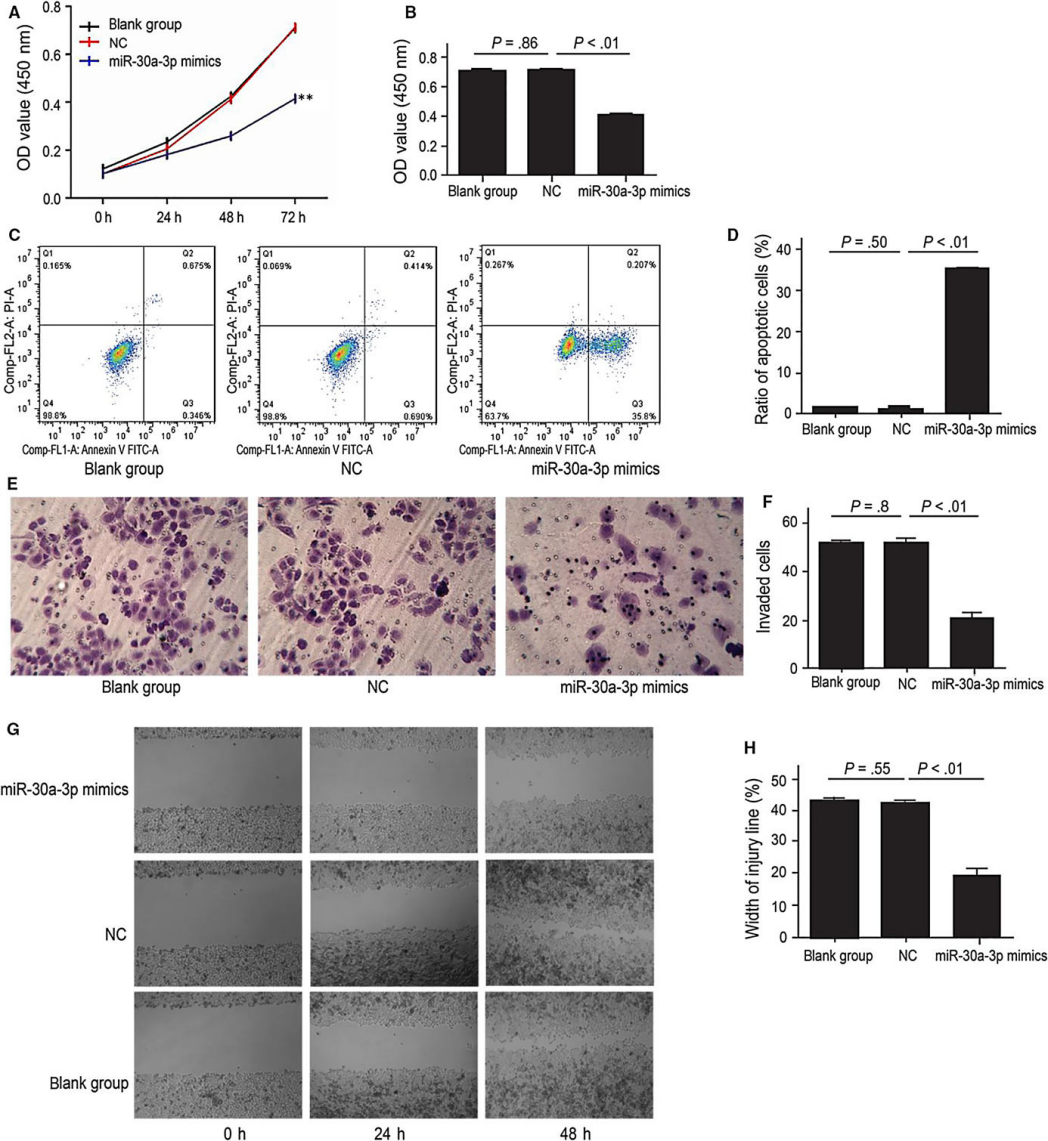
MiR‐30a‐3p can inhibit proliferation and promote apoptosis of BGC823 cells. A, B, CCK8 test showed that upregulation of miR‐30a‐3p reduces proliferation of BGC823 cells (**P < .01). C, D Flow cytometric analysis showed that upregulation of miR‐30a‐3p can increase apoptosis in BGC823 cells (*P* < .01). E, F, Transwell assay showed that upregulation of miR‐30a‐3p can inhibit the invasion ability of BGC823 cells (*P* < .01). G, H, Wound healing assay demonstrated that upregulation of miR‐30a‐3p can inhibit migration of BGC823 cells (*P* < .01)

Interestingly, transfecting BGC823 cells with miR‐30a‐3p mimics resulted in the inhibition of cell proliferation (Figure 5A,B). Furthermore, upregulation of miR‐30a‐3p promoted apoptosis in BGC823 cells (Figure 5C,D) as well as suppressed their migration ability (Figure 5E,F). In the wound healing assay, upregulation of miR‐30a‐3p contributed to slower scratch healing (Figure 5G,H). These results further confirmed that miR‐30a‐3p has a role in gastric cancer cell proliferation, apoptosis and invasion.

**Figure 6:**
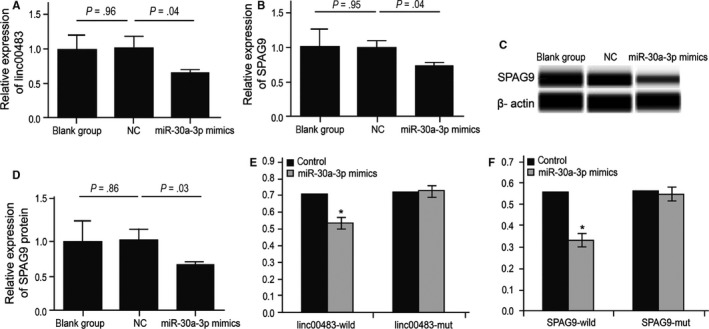
MiR‐30a‐3p can regulate the expression of linc00483 and SPAG9. A, Upregulation of miR‐30a‐3p decreased the expression of linc00483 in BGC823 cells (*P* = .004). B, C, D, Upregulation of miR‐30a‐3p decreased the expression of SPAG9 mRNA and protein in BGC823 cells (*P* = .04, *P* = .03). E, F, BGC823 cells cotransfected with miR‐30a‐3p mimics or control, Renilla luciferase vector pRL‐SV40 and full length linc00483 or SPAG9 3′ UTR luciferase reporter for 48 h. Both firefly luciferase signals were normalized with the Renilla luciferase signals. Results were normalized to those for cells treated with control mRNAs at 100% (*P < .05)

Overexpression of miR‐30a‐3p resulted in decreased expression of linc00483 and both SPAG9 mRNA and protein (*P* = .04, *P* = .04, *P* = .03; Figure 6A‐D). By using the luciferase reporter gene assay, we showed that miR‐30a‐3p can significantly decrease the signal of both wild‐type linc00483 and SPAG9 3′ UTR, while it had no effect on mutant linc00483 and SPAG9 3′ UTR (*P* < .01; Figure 6E,F). These results show that miR‐30a‐3p can specifically bind and negatively regulate linc00483 and SPAG9.

### 3.8 Linc00483, as a ceRNA, upregulates SPAG9 and activates MAPK to contribute to tumour growth in vivo

**Figure 7:**
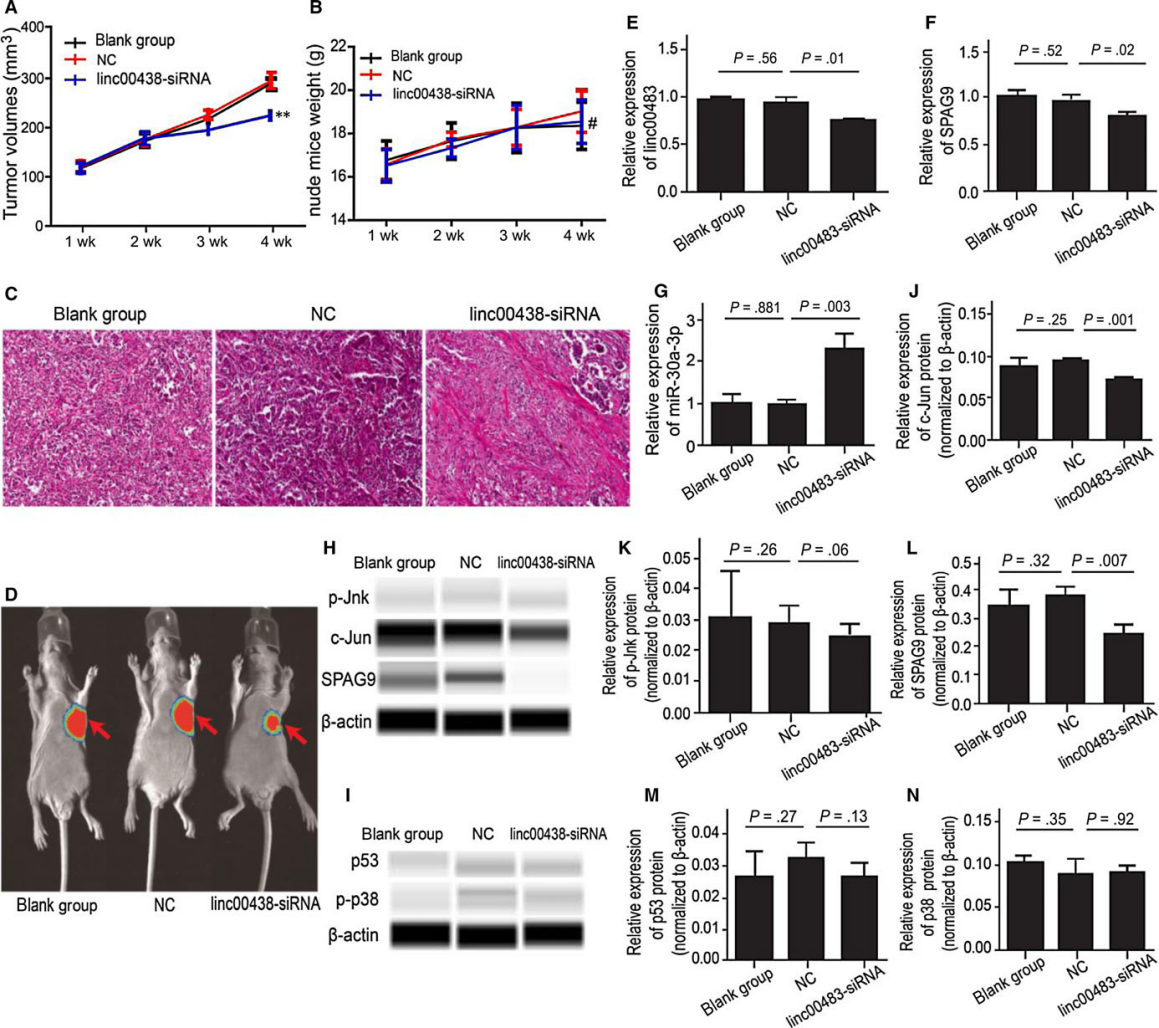
Linc00483 can promote proliferation, inhibit apoptosis and regulate activation of MAPKs in vivo. A, Knockdown of linc00483 can inhibit growth of xenografts in nude mice (**P < .01). B, Knockdown of linc00483 does not impair growth of weight in nude mice (^#^ *P* > .05). C, Knockdown of linc00483 can inhibit cell proliferation as examined by haematoxylin‐eosin staining in nude mice. D, Knockdown of linc00483 can inhibit growth of xenograft as demonstrated by fluorescence intensity analysis in nude mice. E, Suppression of linc00483 can decrease the expression of linc00483 in the xenograft (*P* = .01). F, Suppression of linc00483 can decrease the expression of SPAG9 mRNA in the xenograft (*P* = .02). G, Suppression of linc00483 can increase the expression of miR‐30a‐3p in the xenograft (*P* = .003). H, J, L, Suppression of linc00483 can decrease the protein expression of SPAG9 and c‐Jun in nude mice (*P* = .001 and *P* = .007 respectively). H, I, K, M, N, Suppression of linc00483 did not impair the protein expression of p53, p‐Jnk and p‐p38 in nude mice (*P* = .13, *P* = .06 and *P* = .92 respectively)

To determine the effect of linc00483 on tumorigenesis in vivo, we generated a xenograft model by subcutaneously implanting BGC823 cells in nude mice. Nude mice were randomly divided into 3 groups with 6 mice in each group (linc00483‐siRNA, blank group and NC) and injected, respectively, with linc00483‐shRNA, control vector and NS, and one nude mouse of each group was used for fluorescence intensity analysis of the xenograft model. We observed that suppression of linc00483 resulted in significantly decreased tumour growth compared with the blank group and NC, but no difference in mice weight was observed (Figure 7A,B,D). Histological examination showed that knockdown of linc00483 resulted in lower cell numbers in tumour tissue compared to those in the other groups (Figure 7C). Intriguingly, RT‐qPCR showed that knockdown of linc00483 can markedly decrease SPAG9 mRNA and increase miR‐30a‐3p (*P* = .01, *P* = .02 and *P* = .03, respectively; Figure 7E,F,G). Furthermore, western blotting analysis showed that knockdown of linc00483 dramatically downregulated SPAG9 and c‐Jun protein (*P* = .001, *P* = .007), without significantly affecting p53, p‐Jnk and p‐p38 protein expression (*P* = .13, *P* = .06, *P* = .92, respectively; Figure 7H‐N). These results further confirmed that linc00483 bound miR‐30a‐3p and upregulated SPAG9, which resulted in the activation of MAPKs, leading to proliferation and inhibition of apoptosis.

## 4 DISCUSSION

Although much attention has been given to cancer‐related lncRNAs recently, only a few lncRNAs have been characterized in terms of their exact roles in malignant tumours.^26^, ^27^, ^28^ The lncRNAs GClnc1, HOTAIR, RMRP and GAPLINC have been characterized in gastric cancer.^20^, ^29^, ^30^, ^31^ However, these lncRNAs represent the tip of the iceberg, and the possible roles of many lncRNAs have yet to be determined in gastric cancer.

In our previous study, we reported aberrant expression lncRNAs via an ArrayStar microarray in gastric cancer.^24^ From these lncRNAs, we selected a new upregulated linc00483 for examination of its role in gastric cancer models in vitro and in vivo. In this study, a higher linc00483 level was associated with greater tumour size and tumour invasion and was inversely correlated with survival time in patients with gastric cancer. Furthermore, ROC analysis showed that linc00483 may be a potential gastric cancer biomarker. Indeed, our results showed that linc00483 possesses oncogenic activity with multiple effects on gastric cancer cell proliferation, apoptosis, migration and invasion.

The relationships between lncRNAs and protein‐coding RNAs identified via the microarray study suggest that linc00483 is associated with SPAG9 in gastric cancer. SPAG9 is a scaffold protein in the MAPK pathway that activates this pathway in tumorigenesis.^32^ Our results indicated that SPAG9 mRNA expression was upregulated in gastric cancer tissues, and higher levels of SPAG9 mRNA were associated with shorter survival time and increased migration and invasion in gastric cancer patients, which was consistent with the previously described role of SPAG9 in renal cell carcinoma migration, invasion and poor prognosis.^32^ Moreover, in our study, SPAG9 protein and the downstream effectors of MAPKs c‐Jun and p53 were upregulated, which further confirmed that the MAPK pathway is activated in gastric cancer.^33^ Furthermore, knockdown of linc00483 inhibited proliferation and promoted apoptosis among gastric cancer cells, while decreasing SPAG9 and c‐Jun protein expression in vitro and in vivo. Therefore, we can conclude that linc00483 mediates the function of SPAG9 and MAPKs in promoting proliferation and inhibiting apoptosis in gastric cancer.

Bioinformatic analysis revealed that linc00483 shares miR‐30a‐3p MREs with SPAG9 3′ UTR. Promisingly, we found miR‐30a‐3p was downregulated in gastric cancer tissues, and higher levels of miR‐30a‐3p were associated with longer survival time in gastric cancer patients, which was consistent with its previously reported function in hepatocellular carcinoma.^34^ Consistently, ectopic expression of miR‐30a‐3p inhibited proliferation and promoted apoptosis, while decreasing linc00483 and SPAG9 mRNA and protein expression in vitro. We further determined that miR‐30a‐3p can specifically bind to linc00483 and the SPAG9 3′ UTR. Therefore, our results confirm that linc00483 regulates SPAG9 mRNA expression by competing for miR‐30a‐3p, which targets SPAG9 mRNA for degradation.

Furthermore, we found that linc00483 promoted proliferation and inhibited apoptosis in xenograft tumour model in nude mice. Our results showed that knockdown of linc00483 inhibited tumour growth, increased miR‐30a‐3p levels, and decreased both SPAG9 and c‐Jun protein levels in the xenograft model. These findings further confirm that linc00483 can act as a ceRNA sponge for miR‐30a‐3p, which then leads to upregulation of SPAG9 and to MAPK signaling activation, contributing to tumour cell proliferation and apoptosis inhibition in gastric cancer.

In conclusion, in this study we have identified a novel linc00483 that is upregulated in gastric cancer and correlates with patients’ prognosis. Our results revealed cross‐talk between linc00483 and SPAG9 mRNA via competition for miR‐30a‐3p, thus enhancing the expression of the miR‐30a‐3p target gene SPAG9, and then activating MAPKs to promote proliferation and inhibit apoptosis among gastric cancer cells. Based on these findings, linc00483 may be considered a potential biomarker and therapeutic target in future gastric cancer studies.

## CONFLICT OF INTERESTS

The authors declare no potential conflicts of interest.
