# Supplementary material for: Linc00483 as ceRNA regulates proliferation and apoptosis through activating MAPKs in gastric cancer
Source: J Cell Mol Med. 2018 May 15;22(8):3875–86. doi: 10.1111/jcmm.13661 (PMC6050491; doi:10.1111/jcmm.13661)

Table S1 Aberrant expression of lncRNAs

| **Gene Symbol** | **Regulation** | **FC** | ***p*** |  |
| --- | --- | --- | --- | --- |
| LINC00483 | Up | 207.6425 | 4.67E-05 |  |
| XLOC_006390 | Up | 108.85076 | 0.00318685 |  |
| RP11-462G2.1 | Up | 81.69372 | 0.005015885 |  |
| RP11-150O12.3 | Up | 70.630486 | 0.000896916 |  |
| RP11-89K21.1 | Up | 44.67928 | 0.002183593 |  |
| CTD-2354A18.1 | Down | 163.69966 | 1.73E-13 |  |
| RP11-400N13.2 | Down | 154.65607 | 3.15E-08 |  |
| RP11-231N3.1 | Down | 74.84269 | 5.29E-07 |  |
| CALB2 | Down | 71.95296 | 0.010346811 |  |
| AC002066.1 | Down | 65.5398 | 0.000196508 |  |

**Table S**2 Correlation between linc00483 expression and clinicopathological characteristics of gastric cancer patients.

| **Characteristic** | **Total** | **linc00483 expression (mean±SD)** | ***p* value** |
| --- | --- | --- | --- |
| **Age (years)** | |  |  |
| **≥60** | 25 | 2.66±1.68 | 0.47 |
| **<60** | 23 | 3.03±1.92 |  |
| **Gender** |  |  |  |
| **male** | 24 | 2.75±1.69 | 0.73 |
| **female** | 24 | 2.93±1.91 |  |
| **Diameter (cm)** | |  |  |
| **≥5** | 22 | 3.73±1.96 | 0.0008 |
| **<5** | 26 | 1.01±1.21 |  |
| **Pathological differentiation** | |  |  |
| **high-moderate differentiation** | 25 | 2.19±1.12 | 0.001 |
| **poor-undifferentiated differentiation** | 23 | 3.54±2.11 |  |
| **TNM** |  |  |  |
| **I-II** | 29 | 2.27±1.07 | 0.005 |
| **III- IV** | 19 | 3.70±2.29 |  |
| **Lymph node metastases** | |  |  |
| **yes** | 25 | 3.51±1.73 | 0.005 |
| **No** | 23 | 2.10±1.58 |  |
| **Distant metastases** |  |  |  |
| **yes** | 15 | 4.69±1.8 | 0 |
| **No** | 33 | 1.99±0.98 |  |

**Table S3 P**rimer sequences

| ID | Primer | 5’ to 3’ sequence |
| --- | --- | --- |
| SPAG9 | Sense | TTGGCAGTTTTTCAGCCGAC |
| SPAG9 | Antisense | ATGAGACGTGGGTGCATTGT |
| Linc00483 | Sense | GCTGAACCGGAACAGGACAT |
| Linc00483 | Antisense | CCAGTTCACAGCAACTCACG |
| GAPDH | Sense | AACTTTGGTATCGTGGAAGGAC |
| GAPDH | Antisense | GCAGGGATGATGTTCTGGAG |
| U6 | Sense | CTCGCTTCGGCAGCACA |
| U6 | Antisense | AACGCTTCACGAATTTGCGT |

**Figure S1 Expression of aberrant lncRNAs in gastric cancer.** XLOC-006390, RP11-462G2.1, RP11-150O12.3, RP11-89K21.1, CTD-2354A18.1, RP11-400N13.2 and AC002066.1 showed no significant difference in expression between GC tissues and paired normal gastric tissues (*p*=0.14, *p*=0.32, *p*=0.84, *p*=0.24, *p*=0.34, *p*= 0.49 and *p*=0.114, respectively). Linc00483 and RP11-231N3.1 were overexpressed in GC tissues (*p*<0.01 and *p*=0.018, respectively). CALB2 was downregulated in GC tissues (*p*<0.01).


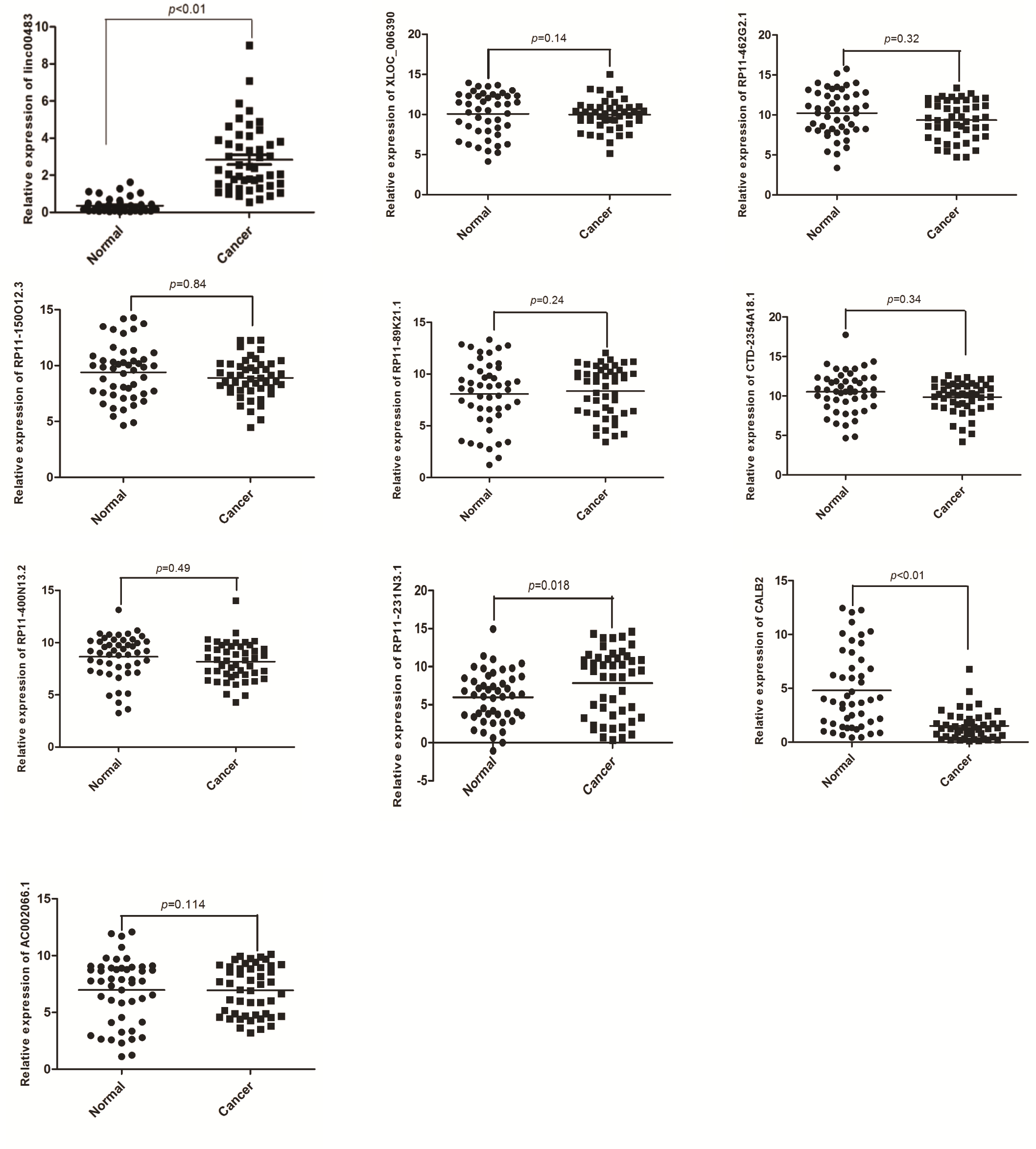


**Figure S2 Expression of aberrant lncRNAs in gastric cancer cells**. **p*<0.05, ***p*<0.01, #*p*>0.05


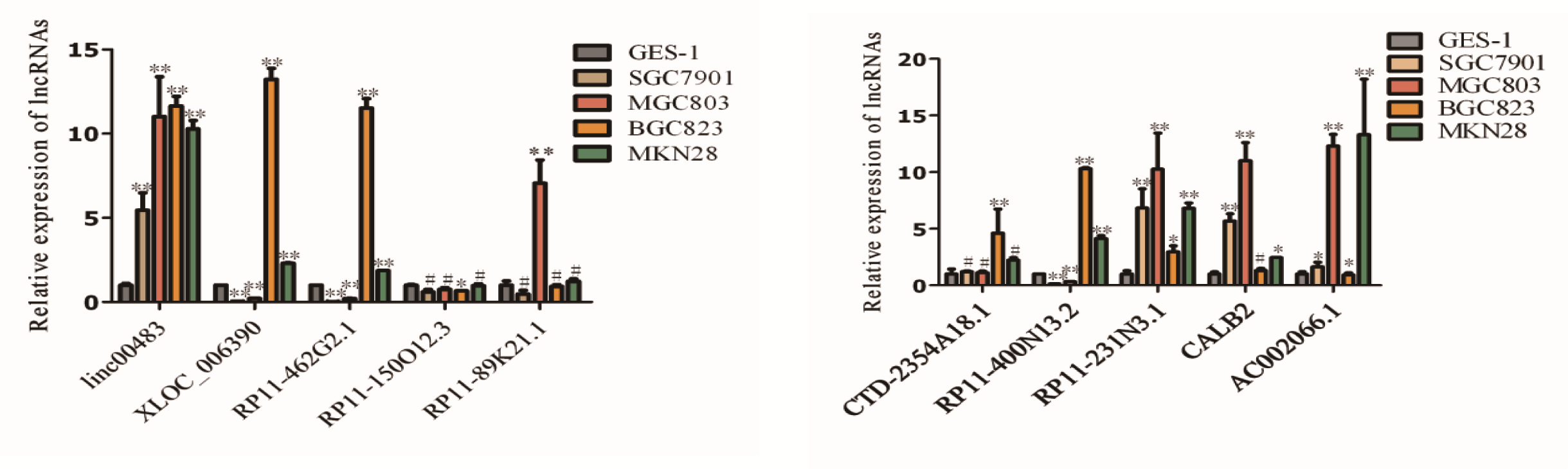


**Figure S3 Annotation of linc00483 in UCSC.** Linc00483 was shown to be a long intergenic non-protein coding RNA, located on choromosome 17 and containing 825 bp.


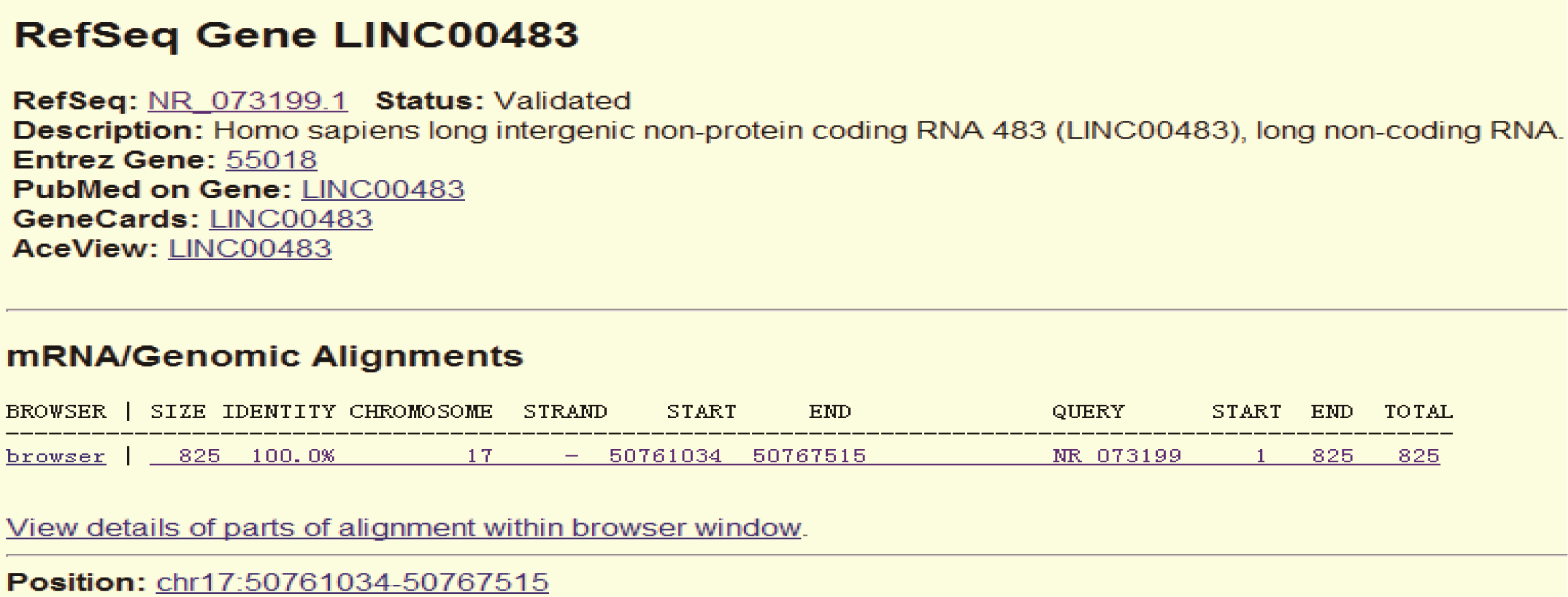


**Figure S4 Linc00483 up-regulation in gastric cancer tissues.** A, Relative expression of linc00483 in 48 paired gastric cancer and normal gastric tissues (*p*<0.01). B, Survival time after surgery was compared between gastric cancer patients with high and low linc00483 expression by Mantel-Cox test (*p*=0.0088). C, ROC for linc00483 expression level as a biomarker for predicting gastric cancer (AUC=0.959; *p*<0.01).


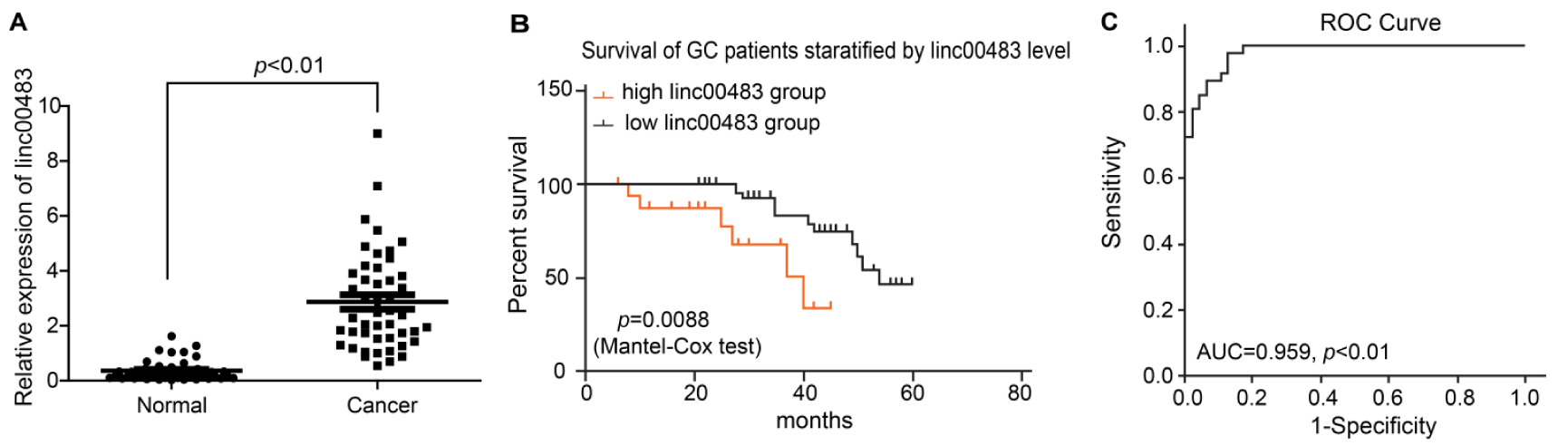


**Figure S5 Efficiency of linc00483-siRNA in BGC823.** Linc00483-Homo-313 was more effective than Linc00483-Homo-364, Linc00483-Homo-417 and Linc00483-Homo-457. **p*<0.05, ***p*<0.01.


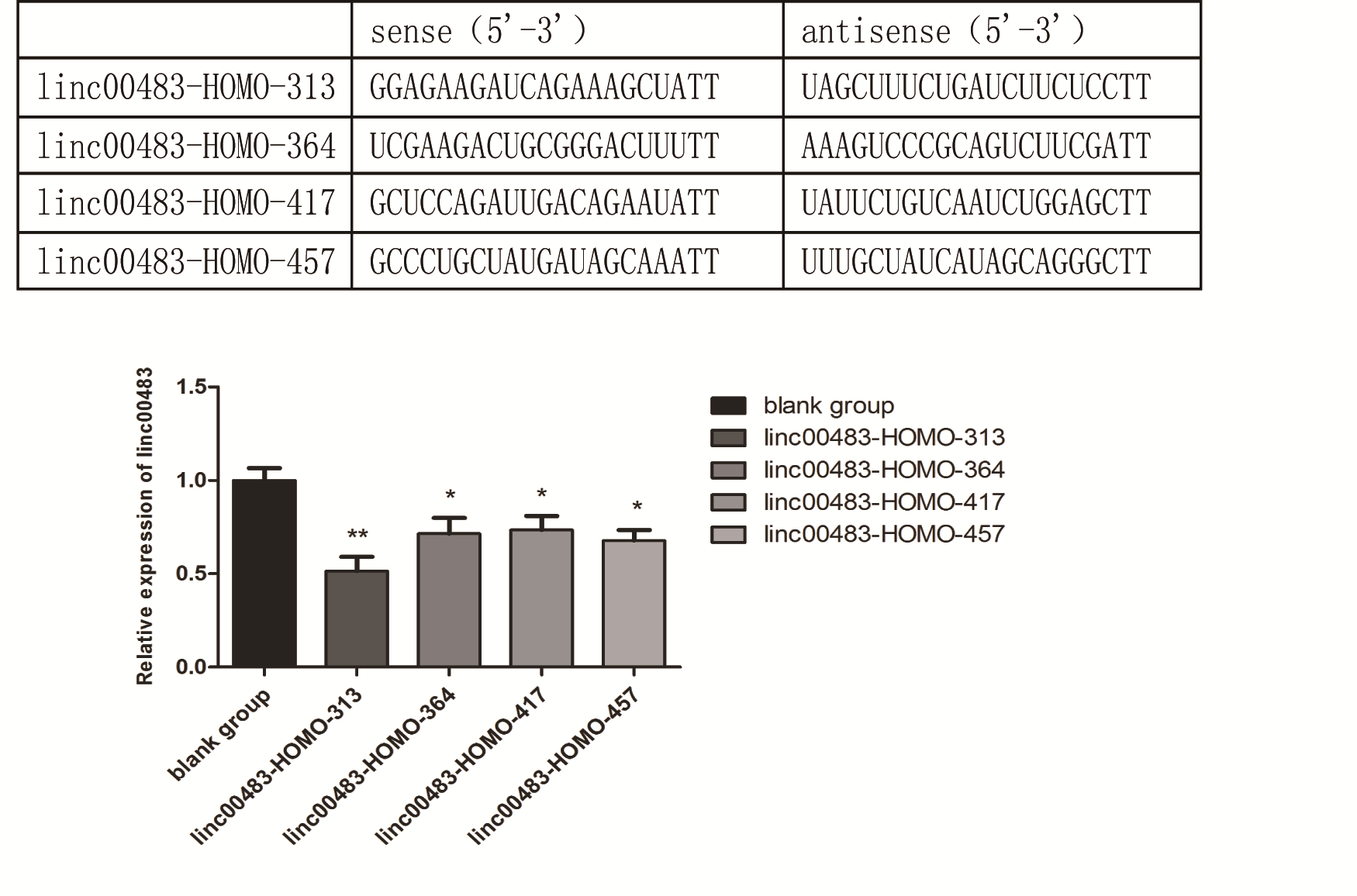


**Figure S6 mRNA expression by microarray in linc00483-siRNA and control group. A** Cluster analysis showed lncRNA different expression (red indicates overexpression and green indicates reduced expression). B Scatterplot showing differences in lncRNA expression; greater dispersion indicates larger differences in expression. C GO analysis demonstrated differentially expressed genes were enriched in gastric cancer. D KEGG pathways indicated that differentially expressed genes were involved in MAPK pathways.


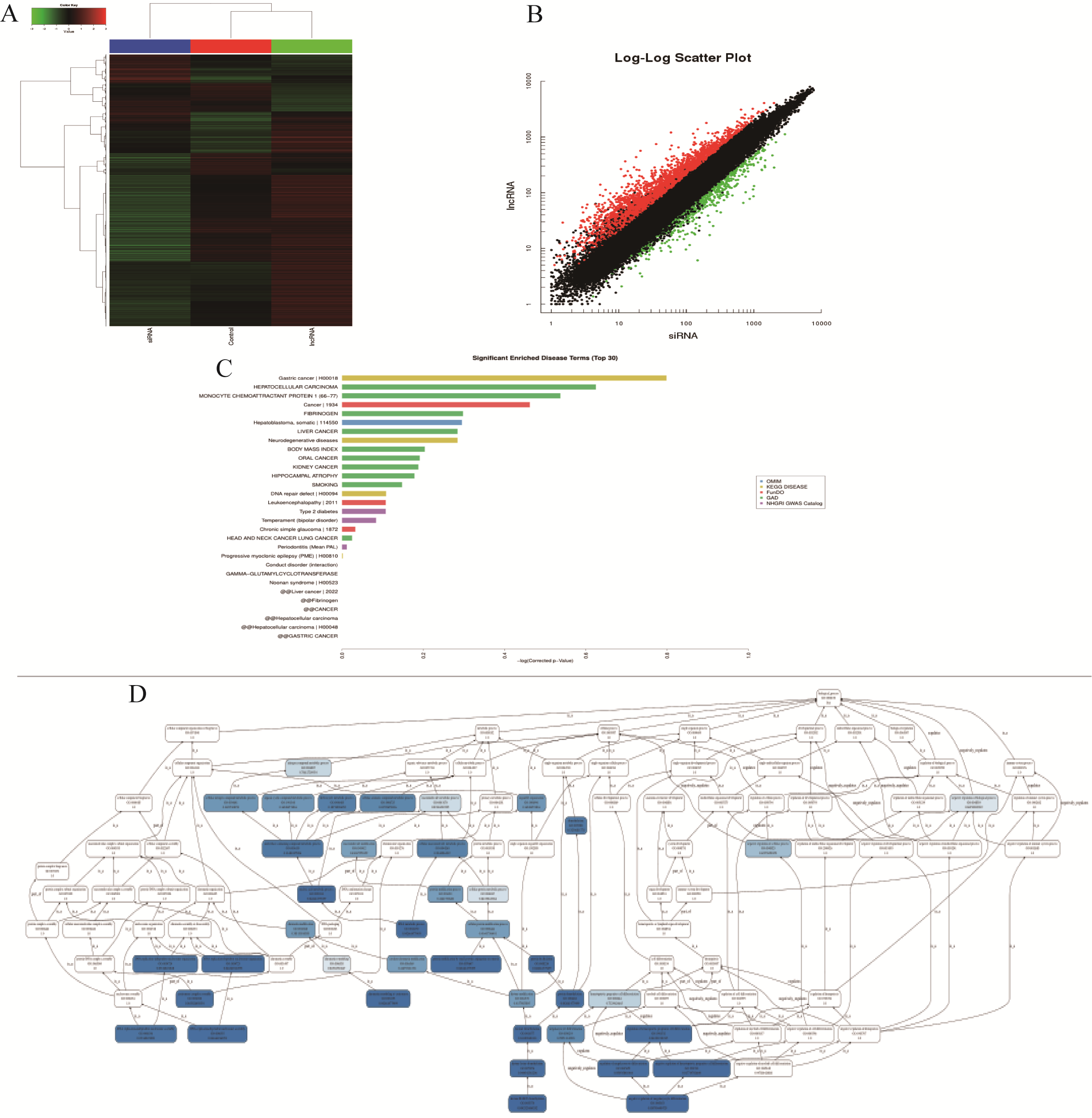


**Figure S7 SPAG9 mRNA over-regulation in gastric cancer tissues.** A, Bioinformatic analysis showed that linc00483 and the SPAG9 gene are located in the same chromosome. B, Relative expression of SPAG9 mRNA in 48 paired gastric cancer and normal gastric tissues showed that SPAG9 mRNA expression was significantly higher in gastric cancer tissues than in corresponding normal gastric tissues (*p*<0.01). C, Survival time of patients after surgery was compared between patients with high or low SPAG9 mRNA expression by Mantel-Cox test, and the survival time of patients with low SPAG9 expression was significantly longer (*p*=0.0027). D, Correlation between linc00483 expression and SPAG9 mRNA based on Pearson correlation analysis showed that linc00483 expression positively correlates with SPAG9 mRNA expression in gastric cancer tissues (R=0.6170, *p*<0.01).


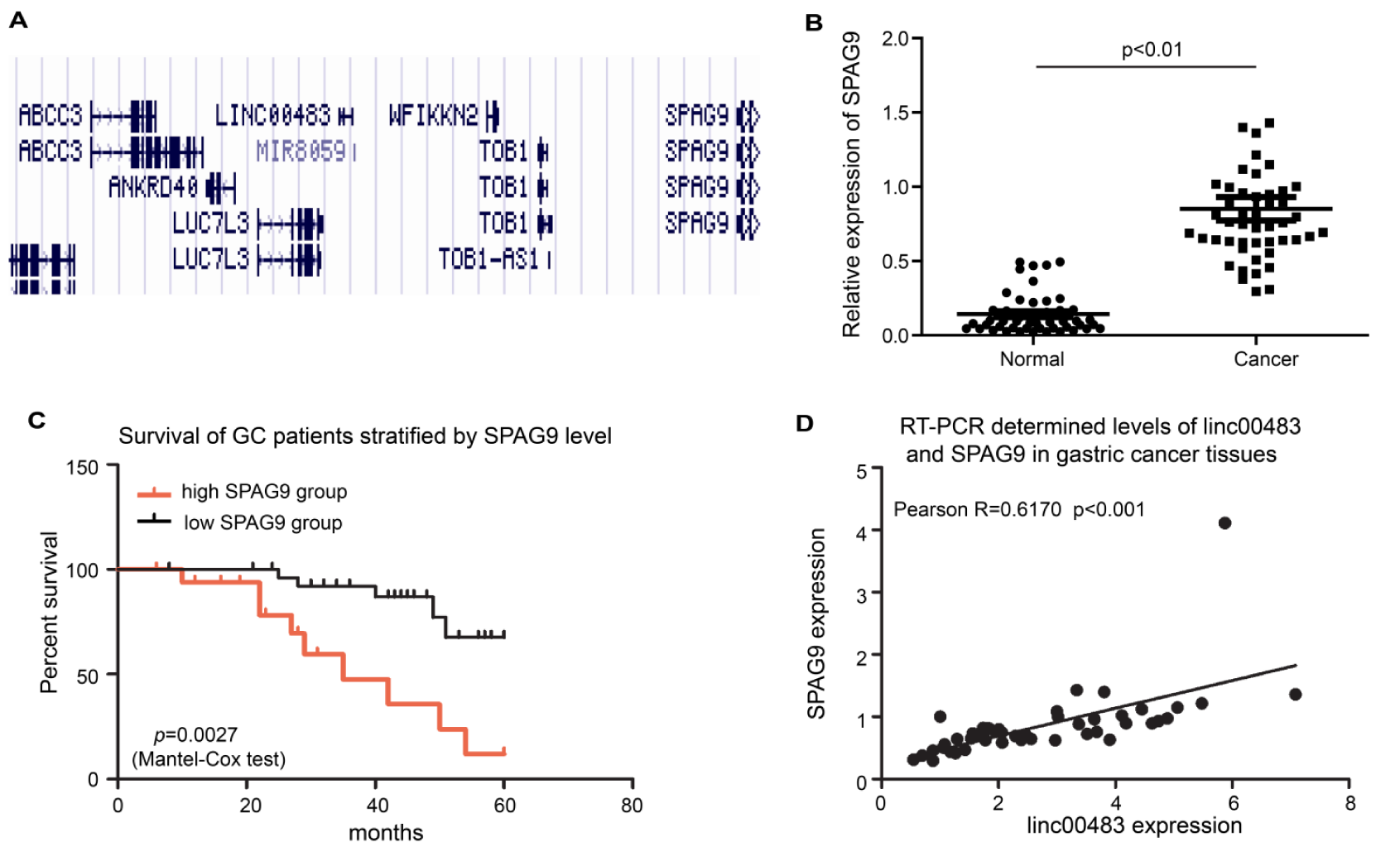


**Figure S8 Original WB images. (1: blank group, 2: NC, 3: linc00483-siRNA).**


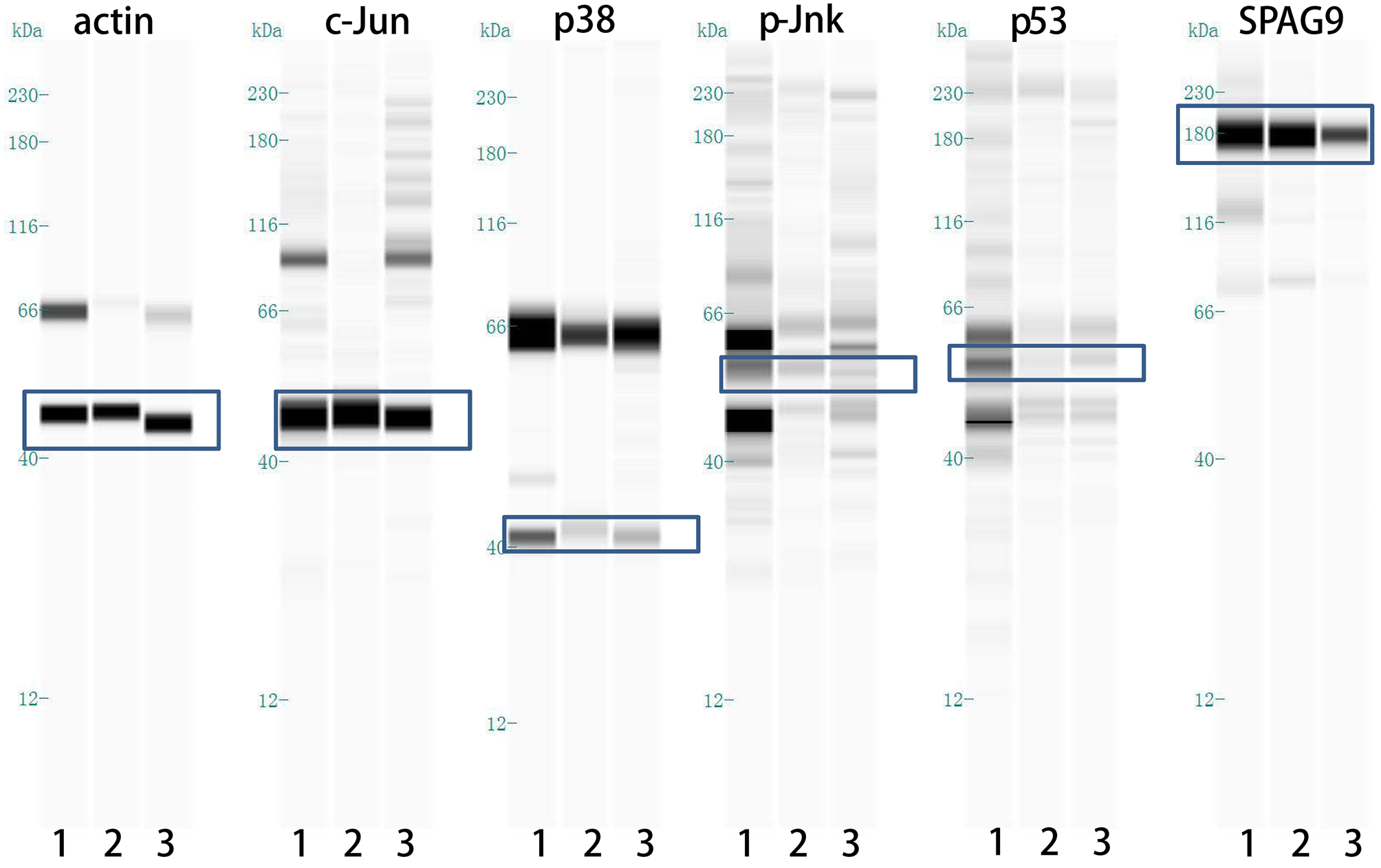

Supplement: Supplementary file 1 [file JCMM-22-3875-s001.doc]
